# Social Media Insights Into Disease Burden in Patients and Caregivers of Myelodysplastic Syndrome: Subcohort Analysis of High-Risk Patients

**DOI:** 10.2196/65460

**Published:** 2025-08-22

**Authors:** Rohit Marwah, Subrat Mishra, Benjamin Gross, Sandra Couturiaux, Rico Calara, Eduardo Jose Sabate Estrella, Cosmina Hogea

**Affiliations:** 1 Definitive Healthcare, 492 Old Connecticut Path Suite 401, Framingham, MA, 01701, United States, 1 5087204224; 2 Gilead Sciences Inc, Foster City, CA, United States

**Keywords:** myelodysplastic syndromes, high-risk MDS, social media listening, natural language processing, patient experience, caregiver perspectives, sentiment analysis, digital health, real-world evidence, health communication, patient-centered insights, information-seeking behavior, unmet needs, machine learning, qualitative data mining

## Abstract

**Background:**

Social media platforms offer valuable insights into patients’ experience, revealing organic conversations that reflect their immediate concerns and needs. Through active listening to lived experiences, we can identify unmet needs and discover the real-world challenges that patients and caregivers face.

**Objective:**

The aim of our study is to develop a reusable framework to collect and analyze evolving social media data, capturing insights into the experiences of individuals with myelodysplastic syndromes (MDS) and higher-risk MDS and their caregivers. The findings can inform the development of appropriate patient support interventions.

**Methods:**

We conducted a structured Google search of English-language websites relevant to MDS from January 1, 2008, to December 31, 2022, using validated URLs and keywords. Data were sourced from MDS-specific platforms to ensure clinical relevance. Contextual embeddings (rather than simple keyword matching) were applied to detect semantically meaningful mentions of “MDS.” Scraping algorithms collected, cleaned, and standardized the data. Posts were classified as originating from patients or caregivers using decision-tree tagging based on contextual summaries. Users were categorized as HR-MDS based on explicit mentions of “high-risk” or by referencing criteria aligned with National Comprehensive Cancer Network guidelines (eg, blast count, transplant, chemotherapy use). Each post was analyzed for major themes and sentiment using a supervised machine learning classifier, while latent topics were identified through a semisupervised model.

**Results:**

We analyzed ~5.5 million words from 42,000 posts across 5500 threads by ~4000 users from the United States, United Kingdom, and Canada. Of the 1249 HR-MDS users identified, 587 (47%) were patients and 662 (53%) were caregivers. Dominant sentiments among HR-MDS users included concern (n=974, 78%), anxiety (n=749, 60%), frustration (n=724, 58%), fear (n=724, 58%), and confusion (n=612, 49%). Concern was the top sentiment among caregivers (n=390, 59%), while anxiety led among patients (n=323, 55%). Key topics included blood counts (n=674, 54%), disease burden (n=537, 43%), quality of life (n=450, 36%), treatment options (n=387, 31%), and disease progression (n=387, 31%). Anxiety was frequently tied to health (n=600, 48%), treatment (n=325, 26%), and the diagnostic process (n=250, 20%). Fear stemmed from complications (n=237, 19%) and progression (n=240, 19%). Confusion about diagnosis and disease understanding was reported by 300 (24%). Information-seeking behaviors revealed user interest in treatment interventions (n=238, 19%) and ongoing research (n=212, 17%).

**Conclusions:**

The application of sophisticated natural language processing techniques demonstrates promise in effectively identifying the emerging complex themes and sentiments experienced by HR-MDS users, thereby highlighting the unmet needs, barriers, and facilitators associated with the disease.

## Introduction

Myelodysplastic syndromes (MDS) are a heterogeneous group of hematologic malignancies marked by clonal hematopoiesis and one or more cytopenias, with a variable clinical course and risk of progression to acute myeloid leukemia (AML) [1]. Risk stratification at diagnosis is outlined by National Comprehensive Cancer Network [2] and European Society for Medical Oncology guidelines [3], with earlier recommendations favoring the Revised International Prognostic Scoring System [4]. Newer approaches increasingly incorporate molecular data, such as the International Prognostic Scoring System – Molecular, now widely adopted in Centers of Excellence [5].

High-intensity treatments for high-risk or very high risk patients improve overall and leukemia-free survival [6,7], including allogeneic hematopoietic cell transplantation [8], intensive chemotherapy [9], or targeted therapies for select patients with actionable mutations [10]. Treatment decisions depend on patient fitness and frailty [9], assessed partly through objective tools like the Eastern Cooperative Oncology Group score [11] and Charlson Comorbidity Index [12], though final determinations remain subjective.

The National Comprehensive Cancer Network guidelines emphasize shared decision-making in the treatment of high-risk patients, involving health care providers, patients, and their families, and aligning care plans with the patient’s personal goals. A critical component of this process is ensuring that patients are well-informed about their risk status, prognosis, available treatment options, and associated risks. In navigating these complex decisions, many patients turn to social media platforms to share their experiences. These unprompted, unbiased narratives offer a valuable source of real-world insights that can be harnessed to generate meaningful real-world evidence.

Social media platforms are increasingly recognized as a rich, evolving source of insight into the lived experiences of patients managing chronic illness [13,14]. These unfiltered, unsolicited narratives provide authentic, real-time reflections of what patients and caregivers are most concerned about—capturing emotional, clinical, and logistical challenges over the course of the disease.

Previous studies have explored the general landscape of patient and caregiver experiences with MDS, but none have focused specifically on the high-risk MDS (HR-MDS) population. Notably, Booth et al [15] and Frank et al [16] have laid foundational work in social media listening for MDS; however, key limitations in scope and methodology left important gaps that our study addresses.

Booth et al [15] examined the disease and treatment experiences of 347 patients with AML or MDS who were ineligible for intensive chemotherapy. Their primary objective was to identify the factors driving treatment decisions, particularly in end-of-life contexts. Using manual qualitative analysis, they categorized themes across five broad domains: humanistic, treatment decision, unmet needs, life milestones, and economic burden. Although their study provided valuable insights, it did not identify patient risk status or stratify findings based on it. Moreover, the analysis was qualitative and manual, limiting scalability and depth.

Similarly, Frank et al [16] mined over 20,000 comments from publicly available web-based forums across 6 countries (United States, United Kingdom, Spain, Canada, France, and China) to identify unmet needs. Their study used a proprietary natural language processing (NLP) engine to identify keywords and semantic themes, forming topic networks to understand discussion patterns. However, like Booth et al, this work also did not isolate the HR-MDS subgroup—an essential component for informing higher-risk disease management and support strategies.

Our study is the first to build on these foundations with a specific focus on the HR-MDS population, combining large-scale, disease-specific social media mining with advanced, reproducible NLP techniques. To reach this high-risk cohort, we first developed a scalable, structured approach to collect, clean, and contextualize MDS-related social media posts. We then applied intrinsic classification methods based on National Comprehensive Cancer Network–aligned clinical markers to accurately identify and separate the HR-MDS population from the broader MDS dataset.

We found that patients in the United States, United Kingdom, and Canada frequently reported negative experiences at the time of diagnosis, particularly concerning provider interactions. Themes included a lack of disease awareness among nonspecialist physicians, inconsistent treatment recommendations, and confusion stemming from unclear diagnostic information—echoing and expanding on findings from Steensma et al [17] and others.

By focusing specifically on HR-MDS and introducing a psychographic thematic framework, our study uncovered deeper, structured insights into the milestones, emotions, and burdens most salient to this underrepresented subgroup. These findings go beyond keyword clustering to reveal how patients and caregivers articulate nuanced concerns, enabling the generation of more targeted real-world evidence.

Our primary objectives were to gather, analyze, and characterize the patient and caregiver journey—specifically for MDS and HR-MDS—using organically expressed experiences on social media and construct a thematic and sentiment-based psychographic model to surface unique insights from posts that mention high risk, either explicitly or through derived contextual inference.

## Methods

### Data Sources

This study is a descriptive exploratory analysis of publicly available posts related to the experiences of patients with MDS and HR-MDS and their caregivers on social media and blog sites from August 2008 through November 2022. Publicly available posts refer to content that was freely accessible online without the need for login credentials, subscriptions, or payments. The search process involved both automated and manual methods. A team of clinical experts conducted manual searches using predefined search terms outlined in Textbox 1, ensuring a comprehensive collection of relevant posts. In addition to manual evaluation, extensive automated web scraping techniques were used to systematically retrieve data. The search was limited to publicly accessible platforms, including patient forums, health-related discussion boards, and blogs where individuals shared their experiences with MDS and HR-MDS. The start of the study period was selected because it corresponds to the label expansion of azacitidine to treat patients with HR-MDS. A preliminary analysis of cumulative posts over time was performed to confirm that there was an adequate number of posts during this time period to complete the study objectives.

Textbox 1. **List of keywords and key search terms.**

**Caregiver perspectives on myeloplastic syndrome (MDS)**

- Caring for MDS
- Caring for myelodysplastic syndrome
- MDS caregiver advocacy group
- MDS caregiver blog
- MDS caregiver discussion
- MDS caregiver experience
- MDS caregiver forum
- MDS caregiver journey
- MDS caregiver story
- MDS caregiver support group
- Myelodysplastic syndrome caregiver advocacy group
- Myelodysplastic syndrome caregiver blog
- Myelodysplastic syndrome caregiver discussion
- Myelodysplastic syndrome caregiver experience
- Myelodysplastic syndrome caregiver forum
- Myelodysplastic syndrome caregiver journey
- Myelodysplastic syndrome caregiver story
- Myelodysplastic syndrome caregiver support group

**High-risk MDS and treatment experiences**

- High-risk MDS (story or experience)
- High risk myelodysplastic syndrome (story or experience)
- MDS bone marrow transplant (story or experience)
- MDS stem cell therapy (story or experience)
- MDS transfusion (story or experience)
- MDS treatment advocacy group
- MDS treatment blog
- MDS treatment discussion
- MDS treatment experience
- MDS treatment forum
- MDS treatment journey
- MDS treatment story
- MDS treatment support group
- Myelodysplastic syndrome bone marrow transplant (story or experience)
- Myelodysplastic syndrome stem cell therapy (story or experience)
- Myelodysplastic syndrome transfusion (story or experience)
- Myelodysplastic syndrome treatment advocacy group
- Myelodysplastic syndrome treatment blog
- Myelodysplastic syndrome treatment discussion
- Myelodysplastic syndrome treatment experience
- Myelodysplastic syndrome treatment forum
- Myelodysplastic syndrome treatment journey
- Myelodysplastic syndrome treatment story
- Myelodysplastic syndrome treatment support group

**MDS advocacy and community engagement**

- MDS advocacy group
- Myelodysplastic syndrome advocacy group

**MDS and related conditions**

- MDS aplastic anemia
- Myelodysplastic syndrome aplastic anemia

**Narratives of patients and survivors of MDS**

- I survived MDS
- I survived myelodysplastic syndrome
- Living with MDS
- Living with myelodysplastic syndrome
- MDS patient advocacy group
- MDS patient blog
- MDS patient discussion
- MDS patient experience
- MDS patient forum
- MDS patient journey
- MDS patient story
- MDS patient support group
- MDS survivor advocacy group
- MDS survivor blog
- MDS survivor discussion
- MDS survivor experience
- MDS survivor forum
- MDS survivor journey
- MDS survivor story
- MDS survivor support group
- Myelodysplastic syndrome patient advocacy group
- Myelodysplastic syndrome patient blog
- Myelodysplastic syndrome patient discussion
- Myelodysplastic syndrome patient experience
- Myelodysplastic syndrome patient forum
- Myelodysplastic syndrome patient journey
- Myelodysplastic syndrome patient story
- Myelodysplastic syndrome patient support group
- Myelodysplastic syndrome survivor advocacy group
- Myelodysplastic syndrome survivor blog
- Myelodysplastic syndrome survivor discussion
- Myelodysplastic syndrome survivor experience
- Myelodysplastic syndrome survivor forum
- Myelodysplastic syndrome survivor journey
- Myelodysplastic syndrome survivor story
- Myelodysplastic syndrome survivor support group
- Surviving MDS

Google Search was the initial screening tool used in an exploratory analysis to identify potential data sources for this study. Search phrases carefully designed to holistically capture the experiences of patients with MDS and their caregivers were used on Google Search to build a complete and vetted list of potential data sources. An exhaustive list of search phrases is provided in Textbox 1. Website relevance was also considered to make sure information was collected from reliable sources and is detailed in Table 1.

**Table 1.** Details of data sources that met the study inclusion criteria and were found during the exploratory analysis.

Source	Title
blog.dana-farber	Putting College – and Field Hockey – on Hold for a Bone...
blog.dana-farber	Making the Best of Things In The Hospital - Blog
blog.dana-farber	Feeling Lucky in an Unlucky Situation | Dana-Farber Cancer...
blog.dana-farber	Battling Cancer: Restructuring and Enjoying Your New Life
blog.dana-farber	Stem Cell Transplant Gives MDS^a^ Patient a Second Chance at...
bonemarrowtransplantexperience.wordpress	Bone Marrow Transplant: My Experiences with MDS and AML^b^
cancer.osu	Patient Story: Mark Althouse | OSUCCC – James
childrenscancer	Joseph’s Story: Surviving Myelodysplastic Syndrome (MDS)
csn.cancer	myelodysplastic - Cancer Survivors Network
emmafightsmds.wordpress	Emma’s fight against MDS – My battle against the blood...
forum.bloodcancer	Anybody here have Myelodysplastic Disorder?
mdandersontlc.libguides	Patient Stories - Leukemia and Myelodysplastic Syndromes...
mdspatientsupport	MDS Patient Stories
myelomabeacon	Treatment-related myelodysplastic syndromes (MDS)
parkway.chop	Secondary Myelodysplastic Syndrome (MDS): Zamiyha’s Story
patientworthy	Living with MDS: Ryan’s Story Part 1 - Patient Worthy
patientworthy	Living with MDS: Ryan’s Story Part 2 - Patient Worthy
rarediseases	Voices of Rare Cancer: Brian’s Story - NORD (National...
aamds	MY 12 y BATTLE WITH MDS - By Jane Biehl PhD
aamds	“I’m Like You” Patient Stories | Aplastic Anemia & MDS International …
aamds	Stories of Hope | Aplastic Anemia & MDS International...
anthonynolan	Emma’s story - Living with MDS as a young woman
bloodresearch.or.kr	An interesting story of a clone - BLOOD RESEARCH
cancerresearchuk	Myelodysplastic syndrome | Cancer Chat
curetoday	In the Mist of My Fear: Experiences With MDS - CURE Magazine
curetoday	The Roller Coaster of MDS - Cure Today
elcaminohealth	Maria’s Story: Myelodysplastic Syndrome (MDS)
leukaemia	Stories - Leukaemia Foundation
leukaemia.au	Diagnosed with MDS, AML, then MDS again
mdanderson	Why a myelodysplastic syndrome patient didn’t settle
mdanderson	How a myelodysplastic syndrome survivor found strength
mdanderson	How art helped my daughter cope with MDS treatment
mdanderson	Spouses face back-to-back hairy cell leukemia and...
mdanderson	Life as a myelodysplastic syndrome survivor: New beginnings
mdanderson	How a clinical trial gave me my life back after MDS and AML
mdanderson	Myelodysplastic syndrome survivor: A targeted therapy clinical...
mdanderson	Myelodysplastic syndrome researcher: Leaps of faith led to my...
mdanderson	Myelodysplastic syndromes clinical trial participant: I don’t...
mdanderson	Myelodysplastic syndrome survivor: A stem cell transplant put...
mdanderson	Myelodysplastic syndrome survivor: Why I joined a clinical trial
mdanderson	Myelodysplastic syndrome: What you need to know
mdanderson	My new normal: Life after myelodysplastic syndrome
mds-foundation	Patient Stories - MDS Foundation
mskcc	Nancie’s Story | Memorial Sloan Kettering Cancer Center
youandmds	George’s story: How did you find out you had MDS?
youandmds	Patient Video - George’s story: How did you find out...
youandmds	Patient Video - George’s story: What has been the biggest...
youandmds	Patient Video - David’s story - You and MDS
youandmds	Abby’s story: What advice do you have for other patients on...
YouTube	Becky’s story: What is your MDS gene mutation... - YouTube
YouTube	Barry’s story: What has been the biggest challenge... - YouTube
YouTube	Patient Success Story | Bone Marrow Transplant | Dr. Rajib De
YouTube	Myelodysplastic Syndrome (MDS) patient discusses her...
YouTube	A Patient’s Story: Myelodysplastic Syndrome - YouTube
YouTube	David’s story: What advice do you have for other patients on...
YouTube	Donna’s story: How has your gene mutation profile... - YouTube
YouTube	Holly’s story: What advice do you have for other MDS patients...
YouTube	Story Corner: Memorie Munson (Caregiver, MDS) - YouTube
YouTube	Aplastic Anemia Patient Story - Mychaela Lovelace - YouTube
YouTube	Story Corner: Mario Rivera and Alison Hines - YouTube
YouTube	David’s story: How did you find out you had MDS? - YouTube
YouTube	249: Azra Raza | Myelodysplastic Syndromes And... - YouTube
YouTube	Max, Sophia and Tom Greb’s Story - YouTube
YouTube	George’s story: What advice do you have for other patients on...
YouTube	Patient Story: Akara from Cambodia - YouTube
YouTube	Story Corner: Lisa Zieske (Aplastic Anemia, MDS, PNH)
YouTube	Barry’s story: How was your MDS-related anemia diagnosed?
YouTube	Luke & Molly’s Story - YouTube
YouTube	Story Corner: Joe Ellenberger (PNH, transplant) - YouTube
YouTube	National MDS Day: Jad Harris’ Story - YouTube
YouTube	Story Corner: Adrienne Torrey and Joe Coffidis (MDS)
YouTube	Myelodysplastic syndrome (MDS) survivor Holly... - YouTube
YouTube	Inspirational Story - Bone Marrow Transplant | Survivor Interview
YouTube	Sharing My Voice: Joan’s Story - YouTube
YouTube	The Caregiver’s Journey: Caring for the Caregiver
YouTube	The Caregiver’s Journey: Exploring Emotions
YouTube	The Caregiver’s Journey: Finding Support

a MDS: myelodysplastic syndrome.

b AML: acute myeloid leukemia.

### Setting

In this study, we conducted searches using keywords associated with MDS across all publicly available sources of information where patients and caregivers were involved in digital conversations. The dataset for our analysis comprised social media posts sourced from publicly accessible websites. Criteria for inclusion encompassed posts featuring a user-identifiable username, distinguishing them as either patients with MDS or caregivers/family members, with content directly related to MDS. Forums had to exhibit discussions pertinent to the primary study objectives and maintain activity with posts dating from 2022 onward. Only English-language posts were assessed. We chose posts from websites where accessibility without registration and consent for third-party data analysis was mandatory. Posts meeting these criteria were considered retrievable for inclusion in our analysis. Once the relevant sites, forums, social channels, and discussions were identified, data extraction algorithms specific to each site were written in R or Python programming languages to extract posts from each site. The data extracted included the username, post text, thread title, date of the post, and URL (in cases where it was possible).

At the time of extraction, all data were deidentified to remove any patient identifiers including but not limited to email addresses; web links to personal social media accounts, websites, or blogs; and telephone numbers. We then applied additional checks to exclude posts with duplicates and non–Unicode Transformation Format-8 characters. We also applied autocorrection on misspellings using the Hunspell package in R.

### Criteria for Anonymization

Social media platforms can present some potential risks around patient privacy, and hence anonymization becomes extremely critical [18]. The proposed anonymization algorithm was designed to safeguard sensitive information within the dataset. It operated as follows. First, if any patient or caregiver names, whether first, last, or both, were identified, they were promptly removed and replaced with the generic label “NAME” to ensure anonymity. Similarly, provider names, in any combination of first, last, or both, were substituted with the standardized term “PROVIDER NAME” to maintain confidentiality. To preserve longitudinal data integrity, usernames were tokenized, thus ensuring that posts from individual users remained coherent over time. Institutional names such as NCI (National Cancer Institute) and Dana Farber were retained within the dataset. Additionally, any therapeutic mentioned by its brand name as listed in Table 2 was converted to its corresponding International Nonproprietary Name. Finally, manufacturer names extracted from the Food and Drug Administration’s National Drug Code Directory were expunged and replaced with the generic designation “PRODUCT MANUFACTURER” to protect proprietary information and maintain anonymity throughout the dataset. Posts containing any mention of, or reference to, an investigational drug or specific clinical trial were not scraped into the data corpus (see Exclusion Criteria section). As an example, the phrase, “my oncologist recommended I look at clinical trials” was included, but a post containing “I decided to start a clinical trial my doctor recommended a trial with a new drug called sabatolimab” was not scraped.

**Table 2.** Brand names converted to International Nonproprietary Names.

Brand	International Nonproprietary Name
Venclexta	venetoclax
Tibsovo	ivosidenib
Xospata	gilteritinib
Velcade	bortezomib
Daurismo	glasdegib
Idhifa	enasidenib
Mylotarg	gemtuzumab ozogamicin
Clolar	clofarabine

The methodology to anonymize names was implemented using the anonymizer package in R, which identified given names, surnames, and titles (eg, Dr Smith).

The methodology to exclude references to clinical trials and investigational agents was programmed into the web scraper and validated using posts on forums for liquid tumors other than AML or MDS. Table 2 shows a subset of possible brand names to demonstrate the methodology. All brand names were mapped to the corresponding International Nonproprietary Name using the national drug code index.

### Data Exclusion

Social media posts were considered eligible for inclusion in this study if they met the following criteria: originating from one of the specified forums, containing one of the listed MDS search terms, and being authored by patients or caregivers of patients with MDS who discussed diagnosis or caregiving for individuals diagnosed with MDS, or the progression of MDS to AML or caregiving for individuals experiencing such progression.

**Figure 1.**
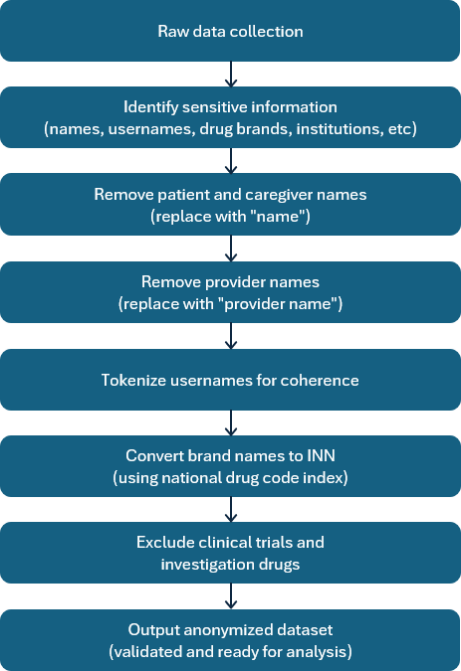
Methodology to map brand names to INN. INN: International Nonproprietary Names.

Posts meeting any of the subsequent criteria were excluded from the study and subsequently removed from the data corpus: posts discussing MDS in a general context without evidence of the author being either a patient or a caregiver; posts explicitly mentioning participation in a clinical trial or discussing a clinical trial, news, and research articles related to MDS lacking patient experiences; and posts related to drug approvals or those primarily focused on content generation without containing the experiences of patients or caregivers. The step-by-step process is briefed in Figure 1.

### Data Management

The data corpus was compiled into an Amazon Web Services database according to the inclusion and exclusion criteria outlined earlier. To ensure the integrity of the dataset, multiple sampling checks were conducted by selecting a subset of posts, which underwent manual evaluation to identify and remove any potentially identifying variables. In addition to manual evaluation, extensive automated web scraping techniques were used, particularly in cases where the primary data source consisted of text data. For data derived from audio or video sources, the content was transcribed into text format, with a subset validated manually. Metadata associated with each data source and post were captured wherever available, including information on discussion threads such as the title, number of posts, and number of views; details about users like tokenized user handle, join date, and location (country and state, if applicable); and post-specific information such as time stamps and replies. All data collected from primary sources were stored electronically as a text corpus along with their respective metadata for further analysis and processing.

### Study Size

An exploratory feasibility analysis of the data sources and data collection criteria described previously identified 3 main forums (MDS Foundation Forums [19], Marrowforums [20], and MDS Patient Support Group [21]) that patients with MDS and caregivers widely use. These forums were the most critical data sources due to the high volume of posts and high user engagement over a significant longitudinal time frame.

Each data source was manually vetted before its inclusion in the study to ensure that the sites were established and legitimate.

The methodology used to assess the quality of sites was based on the site domain. For example, forums, YouTube accounts, and blogs with a verified domain from a cancer center, academic institution, or well-known patient or disease support organizations would be considered trusted sites.

Data sources that meet these criteria are listed below.

### Data Analysis

A web crawler–based search was performed using Google Search to identify websites that had relevant information related to MDS and HR-MDS. After the validation of source URLs, custom scraping algorithms were created to collect all relevant information from these websites/links.

The corpus of text comprising the experiences of patients with MDS and HR-MDS and their caregivers (described in Table 1) was cleaned and normalized in preparation for data analysis. The text was converted into numeric vectors, which preserved the semantics and served as the primary inputs for various algorithms that were used to analyze the data.

Machine learning (ML) algorithms, including topic modeling and unsupervised clustering, were used to detect latent themes in the data automatically. Each latent theme and cluster uncovered by the algorithms was then manually evaluated for the semantic cohesion of members.

NLP was used for data analysis by first topic modeling to identify latent themes, enabling the clustering of documents based on these themes. An ML classifier was then applied to map posts to specific themes, structuring them in a format resembling a patient journey. In the next step, theme segmentation was conducted to categorize and attribute attributes to each theme. This process involved 3 primary approaches: a top-down approach that defined attributes based on overall disease knowledge, including symptoms, disease progression, quality of life, affect, and sentiment; a bottom-up approach that used a word cloud and other aggregates from the data corpus to guide segmentation; and a hybrid approach incorporating elements of both top-down and bottom-up methodologies for a more nuanced categorization of comments.

### HR-MDS Classification

Using NLP and contextualization we identified the mentions of various keywords and keyword combinations to deem a post and the patient and caregiver as part of the higher-risk MDS cohort. A post was considered positive for HR-MDS classification if the post mentioned the use of hypomethylating agents, progression to AML, allogeneic hematopoietic cell transplantation/haploidentical hematopoietic cell transplantation, azacitidine, decitabine, venetoclax, any chemotherapy related to MDS, donor lymphocyte infusion, failure of recombinant erythropoietin to improve anemia, multiple transfusions, stem cell transplantation, blast counts equal to or greater than 20%, and intensive chemotherapy.

### Sample Size and Saturation

Usually, theme generation is directly proportional to the number of posts included; however, at some point, new significant themes stop being generated and this is called the saturation point of the analysis. Saturation was defined as when no new significant themes emerged from the analysis.

### User Classification

Classification algorithms automatically classify each experience’s perspective into that of a patient or caregiver. The automated classification results were reviewed systematically for inaccuracies, and appropriate corrective measures were applied.

Classification was a critical step in the analysis, to correctly identify from the post whether the user is a patient, caregiver, or physician, and it was crucial to tap into the correct themes for each group. Certain key phrases such as “I have MDS,” “I was diagnosed with MDS,” “I am living with MDS,” and “I suffer from MDS” were used to determine the type of user from the posts scraped. This was a semiautonomous step that could only classify the sample partially; some manual interventions were required to classify every post.

### Analysis

Qualitative data analysis was performed for each objective. Posts were first qualitatively analyzed using the R Qualitative Data Analysis package. Theme identification as defined earlier in the Methods section was analyzed against the data corpus for each method until the saturation point was reached. The results of theme analysis for each qualitative data analysis algorithm were analyzed for correct theme identification, number of themes identified, and posts required to reach the saturation point for each theme. Once the best algorithm was identified, the occurrence of each theme was quantitatively summarized using descriptive statistics. The corpus of text was cleaned and normalized in preparation for data analysis. The text was then converted into numeric vectors, which captured the semantics and served as the primary inputs for various algorithms used to analyze the data.

Following the qualitative data analysis, a classification algorithm was then used to automatically classify each experience’s perspective into that of a patient or caregiver. The automated classification results were reviewed systematically for inaccuracies, and appropriate corrective measures were applied.

We also evaluated the effect of “prolific users” that post frequently or in-depth posts in each forum or across forums. We normalized this outlier impact using statistical methods and analyzed any actions or sentiments other users might take based on these influencers, and how these themes propagate across users.

ML algorithms, including topic modeling and unsupervised clustering, were used to detect latent themes in the data automatically. Each latent theme and cluster were then evaluated systematically for the semantic cohesion of topic/cluster members.

### Primary Analysis

Advanced NLP techniques combined with ML methods were deployed to analyze the data corpus comprehensively. Additionally, data visualization techniques, such as word clouds, heat maps, histograms, and network graphs, were used to enhance the analysis process. NLP played a crucial role in discerning whether posts were authored by patients or caregivers, determining the sentiment of the authors, and categorizing each post into one or more broad themes:

1. Clinical: Posts addressing patient conditions, diagnoses, disease progression, and monitoring.
2. Diet and lifestyle: Posts focusing on dietary adjustments and day-to-day challenges associated with living with MDS.
3. Education and logistics: Posts centered around understanding the disease and available care options.
4. Emotional: Posts providing or seeking emotional support among patients or between patients and caregivers.
5. Physical: Posts concerning the management of disease symptoms and treatment-related side effects.
6. Transplants: Posts discussing benefits, risks, and personal experiences with transplants.
7. Treatments: Posts related to inquiries or concerns about treatments, including treatment cycles, regimens, decisions, transfusions, and other palliative treatments. This category was further divided into “disease burden” for patients and “human burden” for caregivers.

Moreover, topic modeling and clustering algorithms were used to uncover additional broad themes solely from the data, leading to the segmentation of posts into more detailed subthemes. Posts mapped to various themes and subthemes underwent further analysis to reveal insights regarding the disease journey, treatment or care gaps, unmet needs, disease knowledge, and the stage or phase of the disease journey. Each broad theme and subtheme resulting from the analyses was categorized as actionable or nonactionable and reported accordingly.

### Determination of the Author

The author of each post could either be the patient himself/herself, a caregiver, or a third actor who is neither of the two. Each post, therefore, was assigned one or more of the following labels: patient and/or caregiver.

The process of categorizing users into two distinct groups—patients and caregivers—based on their posts presented challenges due to the extensive amount of text associated with each user and the prevalence of irrelevant or ambiguous information within the posts. To address this complexity, an innovative classification approach was implemented wherein initially, a classification model was deployed to assign each post with either a “Patient” or “Caregiver” label. Subsequently, a user was classified as either a patient or a caregiver if at least 80% of their posts received the same label from the classification model. In instances where no clear majority (80% or higher) was evident, the posts were systematically analyzed in chronological order, progressing from the earliest to the most recent. This analytical process continued until the model confidently assigned a label (“Patient” or “Caregiver”) to the user.

The data corpus used in this study consisted of a substantial volume of posts, necessitating an automated mechanism to assign each post one or more labels as defined previously. This process unfolded in two stages.

First, a numerical vector representing each post was generated using one or more of the following methodologies:

1. Using a transformer-based large language model such as Bidirectional Encoder Representations from Transformers or a similar alternative model to parse the post and generate a semantic numerical vector representation.
2. Using a word frequency–based numerical representation of the post that did not capture the semantic nuances of the post.

Second, the numerical vector representation of each post was inputted into a multiclass classification model trained using a representative subset of the corpus. This model then automatically assigned a predetermined label to each post, accompanied by a confidence score for each label. Various algorithms were used for the multiclass classification task, including linear classifiers (logistic, Bayesian), support vector machines, decision tree-based classifiers, and ensemble methods (AdaBoost, LightGBM, XGBoost).

The selection of the algorithm was informed by random sample analyses and the categorization of posts by 3 human curators, each reviewing at least 5% of all posts independently. Following individual reviews, the curators convened to reach a consensus on the categorization. The model exhibiting the highest sensitivity and specificity in comparison to the final curation was ultimately chosen.

### Theme Assignment

Each post was mapped to several themes in a theme hierarchy. These themes were organized in an inverted hierarchical tree composed of 5 high-level “root” themes, with more detailed themes mapped as branches for 1 or more roots.

The major themes in the hierarchy were as follows:

1. Disease burden.
2. Treatment decision.
3. Unmet needs.
4. Life milestones.
5. Logistic burden.

A combination of two approaches was used for the theme assignment. In top-down analysis, each post was mapped to one of the predetermined major themes documented above. This is essentially a supervised classification ML problem. Bottom-up analysis, which is a complementary approach to top-down analysis, was used to uncover hidden or latent themes in the corpus. Each post was algorithmically assigned to one of several latent buckets. Each latent bucket was analyzed by a subject matter expert. To perform this analysis, each post was transformed into representation vectors. Following that, the numerical vector representations of the posts were used as input to a range of unsupervised ML algorithms, which are elaborated upon later to unveil latent themes. We used latent Dirichlet analysis and latent semantic analysis for topic modeling. We also used unsupervised clustering algorithms including K-means clustering, spectral clustering, agglomerative clustering, DBSCAN, and OPTICS.

Each latent theme discovered during the bottom-up approach was either mapped to one of the major themes as a subtheme or used to expand the list of major themes.

Each major/minor theme in the hierarchy of themes that is an outcome of this multidimensional approach was subsequently categorized and reported as actionable or nonactionable.

At the heart of the primary objectives of this study is identifying themes that are important to patients with MDS and HR-MDS and caregivers. Themes were identified by applying topic modeling and unsupervised clustering to posts and blog articles in the cleaned and normalized data corpus. Themes were scored by their prevalence, that is, the number of unique discussions in the data corpus that mention a theme. Once scored, themes were categorized as minor or major by applying a threshold to the prevalence score. Themes were also categorized as actionable or nonactionable. An actionable theme is one that identifies a gap or need that can be addressed, whereas a nonactionable theme would typically be emotional. Furthermore, a network of themes was constructed by connecting two themes if they are co-mentioned in the same discussion, the strength of the connection being the number of such co-mentions.

### Secondary Analyses

Every post captured during the process of data collection was collected with a date stamp. Each date stamp was converted to the month and year of the post and was used to determine pre–COVID-19 activity or post–COVID-19 activity. A matched pre–COVID-19 period included posts from January 2018 through February 2020. All posts from March 2020 were included in the post–COVID-19 period. Most of the analysis that was performed on the complete data corpus was also conducted during these two time periods to determine changes in activity, behaviors, and perception of patients and their caregivers.

One of the secondary objectives was to determine themes and insights from a very specific group of patients who either in their post mention high-risk or are deemed high-risk due to the presence of certain high-risk treatments or procedures. These treatments and procedures were determined using the National Comprehensive Cancer Network guidelines for the HR-MDS population and were used as surrogates to tag a post as originating from high-risk versus low-risk patients.

To address the secondary objective of elucidating the experiences of patients with HR-MDS and their caregivers, we first used NLP to automatically label each discussion in the data corpus as “low,” “intermediate,” “high,” or “very high” or unknown risk.

Each data source underwent a manual evaluation to ensure the quality and relevance of the data sources considered for the study. The diverse formats of the data sources required distinct criteria tailored to each type of resource. For each data source, at least one or more of the following parameters were used for evaluation: the reputation of the domain hosting the resource, the number of users, the longitudinal duration of user engagement, and the number of views and comments (for audio/video content).

### Ethical Considerations

This study used publicly available, deidentified data from web-based forums and social media platforms. No identifiable personal information was collected, stored, or analyzed. In accordance with established ethical guidelines for internet-based research, the data analyzed were in the public domain and posted voluntarily by users with no expectation of privacy. Therefore, institutional review board or ethics committee approval was not required. The study adhered to the ethical principles outlined in the Declaration of Helsinki and followed guidance from the Association of Internet Researchers regarding the responsible use of online data for research purposes.

## Results

### Overview

A substantial dataset was amassed, comprising roughly 5.5 million words extracted from 42,000 posts distributed across 5500 threads, involving approximately 4000 users (Table 3). Our algorithms identified 1960 (49%) of these users as patients, 1680 (42%) as caregivers, and 360 (9%) as others, primarily hailing from the United States, United Kingdom, and Canada. Among the identified users, 1249 were classified as HR-MDS users, with 588 (47%) being patients and 661 (53%) caregivers.

**Figure 2.**
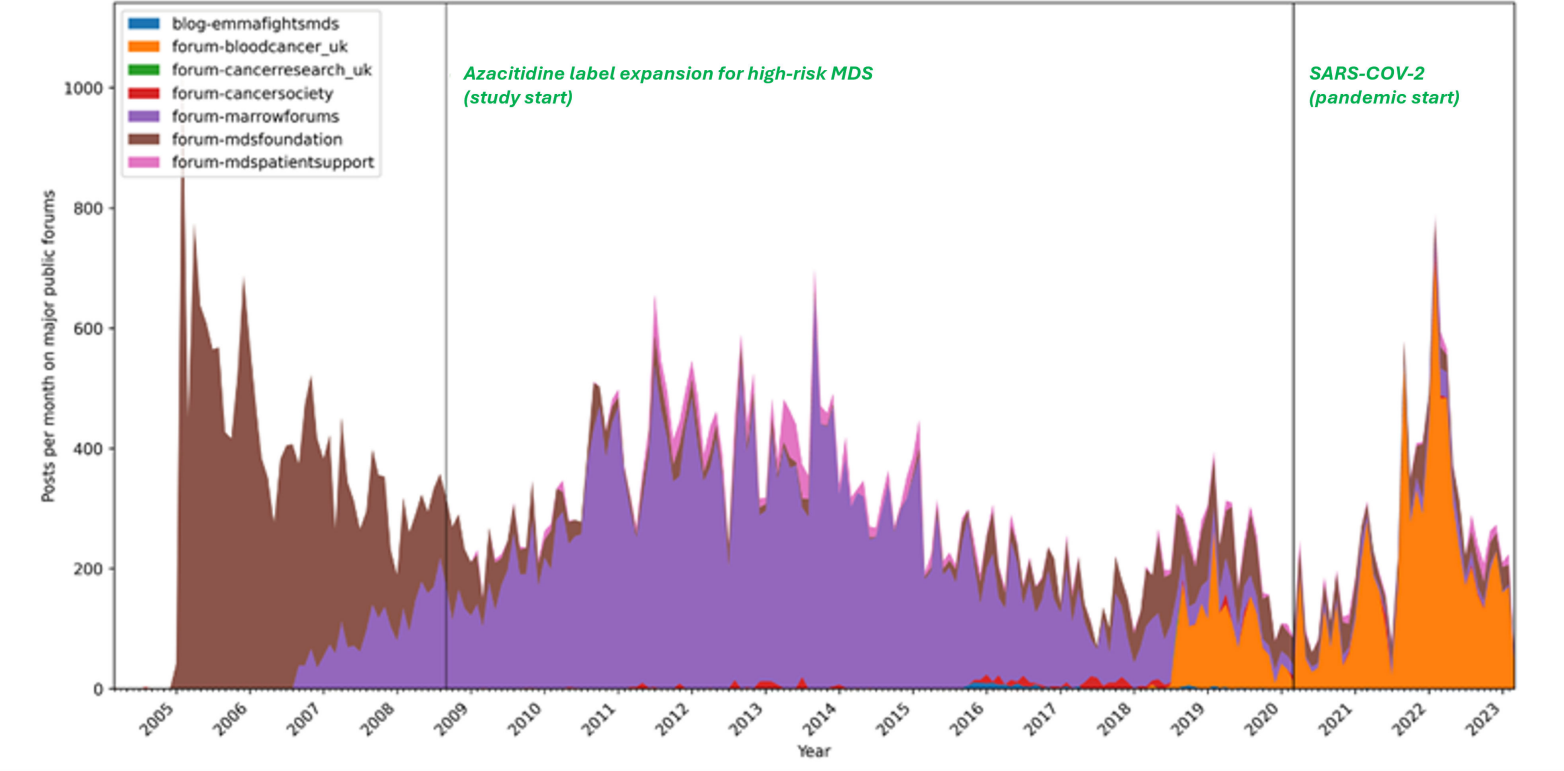
Post frequency of conversation from various sources mapped on a yearly scale from 2005 to 2023. MDS: myelodysplastic syndrome.

Approximately 30 distinct sites were scrutinized for pertinent textual and video content, revealing 3 prominent forum sites that dominated the discourse: MDS Foundation Forums [19], Marrowforums [20], and MDS Patient Support Group [21] (Figure 2).

**Table 3.** Volume of posts found during the exploratory analysis of data sources (August 2008 to December 2022).

Source	Posts	Threads	Users
mds-foundation.org	6834	1426	1379
marrow forums	32,009	3722	1847
Mdspatientsupport.uk	2896	392	199
Total	41,739^a^	5540	3425

a Over 60,000 posts were identified, of which ~42,000 were within this study period (August 2008 to December 2022). The start date was selected based on VIDAZA label expansion for high-risk myelodysplastic syndrome in August 2008.

Analysis of the data suggests that a small fraction of superusers (~1%) wield significant influence over the engagement levels of other users. Notably, the support forum for patients with MDS exhibited the highest level of user engagement, quantified by the duration of user activity (posting) on the forum, which ranged from 2 days to over 1000 days, with a median of 10 days.

Our findings underscore the consistent presence of patients with MDS and caregivers across various forums. However, recent observations indicate a notable shift in activity levels from publicly accessible and analyzable forums to more private forums.

**Figure 3.**
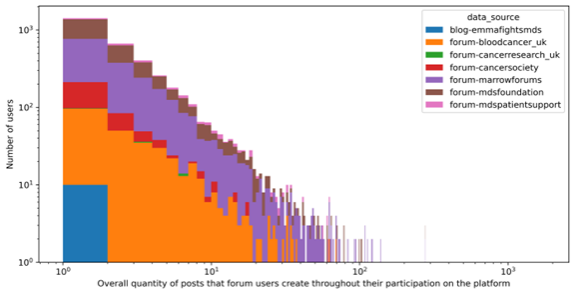
Data analysis revealed that a small group of superusers or influencers significantly impacted other users’ engagement. The “emmafightsmds” forum exhibited a skewed pattern due to the behavior of these superusers, while other forums displayed a more dispersed engagement.

It was noted that most online forums have low user retention rates, with most users disengaging within 2 days. However, Marrow Forums and the MDS Foundation show relatively higher retention rates compared to others. Some superusers are the instigators of these conversations, some reactors react to these posts, and there are passive consumers who consume this information without active participation (Figure 3).

### Emotional Classification

For the emotion classification task, we used a fine-tuned RoBERTa-base model, a transformer-based architecture known for its effectiveness in natural language understanding. The model was fine-tuned using a proprietary, in-house–labeled dataset specifically curated to reflect a diverse and domain-relevant range of emotional expressions. Emotion classification was framed as a multilabel classification task. Prior to training, text inputs were tokenized using RoBERTa’s byte-pair encoding tokenizer and padded to a uniform length. The dataset was randomly split into training (80%) and testing (20%) subsets, maintaining label distribution across splits. Model training was conducted over 100 epochs with a batch size of 16, using the AdamW optimizer and a learning rate of 2e-5. To address class imbalance, we applied a weighted binary cross-entropy loss function. Performance was evaluated on the held-out test set using standard multilabel metrics. The model achieved a macro-averaged *F*_1_-score of 0.78, with precision and recall scores of 0.82 and 0.79, respectively, indicating strong and balanced classification across emotion categories. This approach enabled accurate and interpretable identification of both dominant and subtle emotional signals within the text corpus, supporting robust downstream analyses.

Applying the trained model to our comprehensive analysis of the posts, we discerned 7 major emotional categories. A significant portion of the posts (n=1550, 37%) exhibited a notably neutral emotional tone. Following closely, sadness (n=9240, 22%) and joy (n=8400, 20%) emerged as the second and third most prevalent emotions. Interestingly, 5460 (13%) of the posts conveyed surprise, while the remaining 3367 (8%) depicted emotions revolving around fear, disgust, and anger.

Delving deeper, we endeavored to categorize these primary emotions into finer distinctions. It transpired that 7142 (17%) of the posts resonated with a sense of worry, while 5464 (13%) exuded hopefulness. Summarizing our emotional analysis, posts attributed to patients underscored the emotional weight accompanying the onset of the disease. Patients grappled with fear and harbored anxiety regarding their impending treatment. Numerous posts depicted a common theme: both patients and their caregivers felt ill-prepared for the diagnosis, grappling with confusion stemming from their limited understanding of the disease and available treatment options.

Within the subset of 1249 patients (and their caregivers) identified as having HR-MDS, prevalent sentiments among patients included concern (n=971, 78%), anxiety (n=752, 60%), frustration (n=723, 58%), fear (n=722, 58%), and confusion (n=608, 49%) (Table 4). Notably, concern predominated among caregivers (n=971, 59%), while anxiety was more pronounced among patients (n=752, 55%).

**Table 4.** Predominant sentiments of patients with high-risk MDS. MDS: myelodysplastic syndrome.

Sentiment	Values, n (%)
Concern	971 (78)
Frustration	723 (58)
Anxiety	752 (60)
Confusion	608 (49)
Fear	722 (58)

To take our understanding deeper, we created an organic emotional theme map for each top theme we observed during our data exploration.

**Figure 4.**
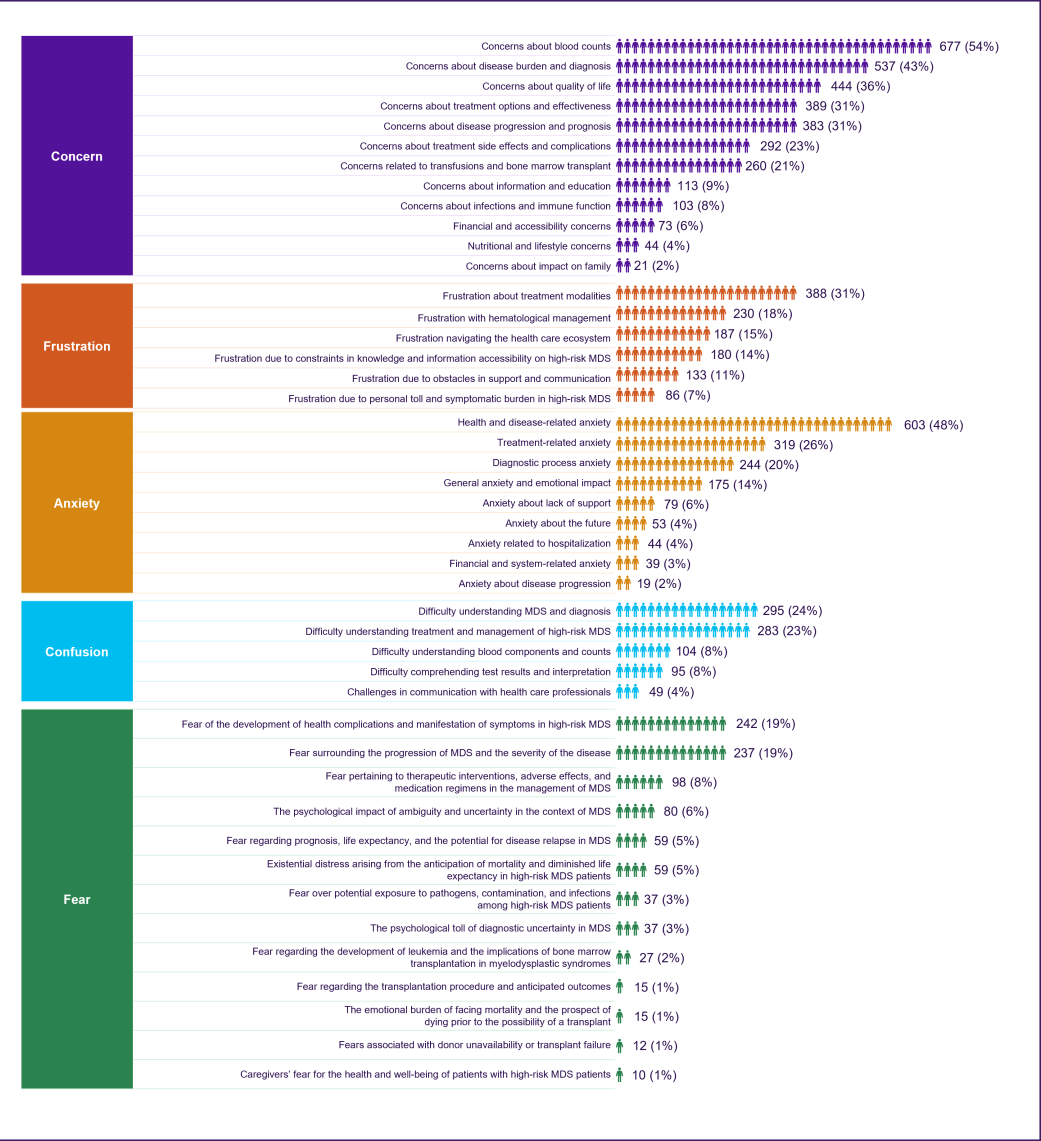
Organic view of subsentiments expressed by patients with high-risk MDS and caregivers for each major sentiment bucket. MDS: myelodysplastic syndrome.

Within the major emotional bucket related to “Concern,” we found that there were some very specific themes related to concerns related to blood counts (n=677, 54%), the burden of the disease (n=537, 43%), quality of life (n=444, 36%), available treatment options and effectiveness (n=389, 31%), and disease progression and prognosis (n=383, 31%). Anxiety related to health and disease (n=603, 48%), treatment (n=319, 26%), and the diagnostic process (n=244, 20%) were also common (Figure 4).

Similarly, within the “frustration” bucket, the top themes observed were related to the frustration of patients and caregivers related to treatment modalities (n=388, 31%), hematological management (n=230, 18%), navigating through the health care system (n=187, 15%), and constraint in knowledge and information accessibility on MDS (n=180, 14%) (Figure 4).

The most common sentiments related to fear were the potential development of health complications and the manifestation of symptoms (n=242, 19%) and the progression and exacerbation of MDS (n=237, 19%). Additionally, confusion was pervasive among participants, with 295 (24%) individuals finding it challenging to comprehend the nuances of MDS and its diagnosis. A systematic analysis of the principal domains for which information is being sought about HR-MDS revealed frequent mentions among users of acquiring information on therapeutic intervention (n=247, 19%) and an interest in ongoing research associated with the disease (n=226, 17%) (Figure 4).

We conducted a more detailed analysis and discovered that within the thematic heading “Frustration related to treatment modalities,” the predominant topics of conversation included discussions on side effects and efficacy, delayed or postponed therapeutic interventions, dependence on transfusions, inadequate pain management, and the economic burden associated with treatment, among others.

**Figure 5.**
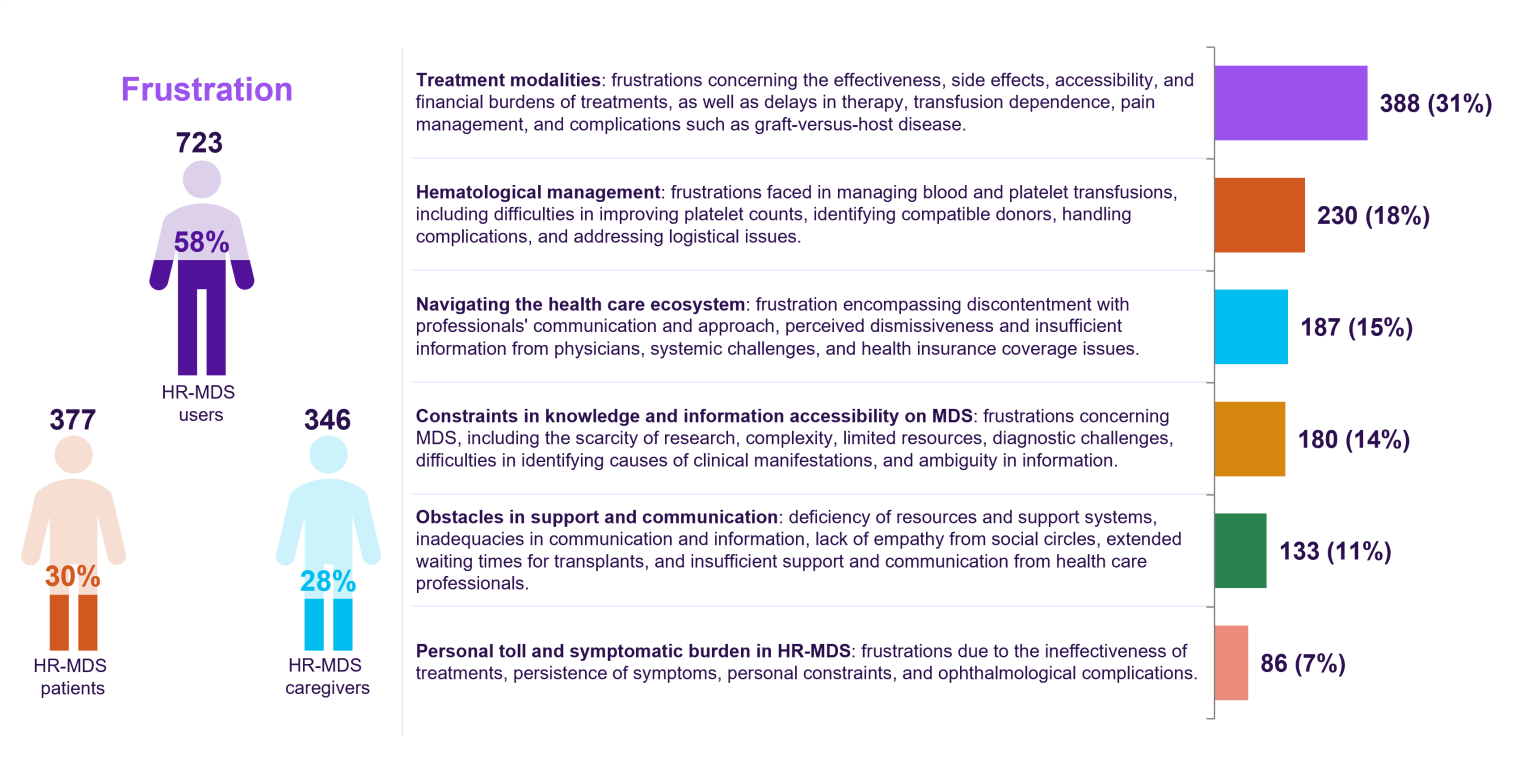
Subtheme-level analysis for the thematic category of frustration. HR-MDS: high-risk myelodysplastic syndrome; MDS: myelodysplastic syndrome.

Within the thematic heading “Navigating the healthcare system,” we observed conversations around discontentment with the physicians and their communication, frustration due to perceived dismissiveness, and a lack of information provided by physicians. Of all the posts, 14% of the posts had topics of conversation related to “Frustration due to constraint in knowledge” and the most common conversations within this thematic bucket were around a lack of understanding of MDS as a disease, heterogeneity of the disease, and ambiguity of the information from physicians. We also observed through these posts that, like with any other disease, patients and caregivers go through severe psychological distress; they experience suffering due to the unknown implications of the available treatment options, complications that come with the disease and side effects, a lack of empathy in social circles, as well as insufficient support and communication from physicians (Figure 5).

## Discussion

### Principal Findings

Our study advances the understanding of patient and caregiver experiences around MDS by uniquely focusing on the HR-MDS subgroup, which has previously been underexplored in the social media literature. Leveraging a large, disease-specific dataset of over 4000 users and 42,000 posts, we used both explicit and intrinsic methods to stratify risk and extract psychographic insights tailored to this high-risk population. This level of granularity, risk-targeted segmentation, and psychographic representation represents a critical gap in prior work and underscores the novelty of our approach.

Social media has emerged as a rich, real-time repository of unfiltered patient and caregiver voices. As noted by Watson et al [13] and Monti et al [14], platforms like online forums and patient communities allow individuals to express their evolving experiences, concerns, and frustrations in ways that are often absent in structured clinical research. These unsolicited narratives can surface emerging patient needs, inform policy decisions, and highlight disparities in care delivery. However, much of the existing research has focused on general MDS populations or aggregated hematological malignancies, without differentiation by risk or disease stage.

For instance, Booth et al [15] conducted a qualitative study on 347 patients with AML or MDS who were ineligible for intensive chemotherapy. Their analysis, although valuable in identifying broad themes such as humanistic burden, treatment decision-making, and unmet needs, relied on manual thematic categorization and did not stratify patients based on risk levels. Although they highlighted critical issues such as the perceived lack of treatment options, confusion over eligibility, and the emotional impact of aging-related treatment biases, the insights remained limited by the sample size and a lack of focus on high-risk patients.

In contrast, our study not only expands the scale by analyzing over 10 times the number of unique voices but also integrates risk identification logic to focus specifically on the HR-MDS subset. This allows us to delve deeper into the specific emotional, diagnostic, and informational hurdles faced by patients and caregivers dealing with a more aggressive form of MDS. We also extend the discussion by capturing sentiment polarity, the temporal evolution of emotions, and user typology (eg, patient vs caregiver), which were not addressed in Booth et al’s work.

Similarly, Frank et al [16] applied proprietary NLP tools to mine over 20,000 comments from multiple countries to identify and categorize the unmet needs of patients with MDS and caregivers. Their study identified broad thematic structures across international patient forums and constructed a semantic network of discussion topics. Although this provided a robust high-level view of concerns such as information gaps, emotional burden, and uncertainty about treatment, it did not differentiate between MDS subtypes or stratify based on disease risk. Furthermore, it focused primarily on surface-level topic identification rather than deep thematic deconstruction or psychographic segmentation.

Our work extends this line of inquiry by offering a comparative framework between general MDS and HR-MDS user experiences. We identified a range of nuanced concerns that disproportionately affect the HR-MDS subgroup, including miscommunication with health care providers, conflicting diagnostic guidance, lack of specialist referral, and frustration stemming from perceived clinical inertia. Notably, patients consistently expressed concerns over the variability in physician expertise, especially when care was delivered outside of MDS Centers of Excellence. This aligns with prior observations by Steensma et al [17] on systemic disparities in MDS care but offers contemporaneous, patient-voiced validation from organic social media data.

Additionally, our study offers an analytical framework that structures the high-volume, high-variability data of social media into interpretable patient journeys, complete with milestone mapping, emotion trajectories, and insight categorization. By doing so, we provide both qualitative richness and quantitative depth—bridging the gap between anecdotal experience and real-world evidence generation. The inclusion of caregivers’ voices further strengthens our findings, illustrating the parallel emotional burden, informational challenges, and advocacy efforts undertaken by families navigating HR-MDS.

Although our study offers valuable insights, it is subject to certain limitations. Social media data may introduce selection bias, as digitally active, younger, or more vocal individuals may be overrepresented. The inferred risk status, though carefully derived, lacks clinical validation and may lead to occasional misclassification. Language nuances and unstructured data also pose interpretive challenges in sentiment and thematic analysis. Although multiple validation steps were used to mitigate these issues, the findings should be viewed as directional and complementary to traditional research methods.

In summary, while previous studies have established the utility of social media in capturing patient sentiment and unmet needs in MDS broadly, our study is the first to systematically focus on the HR-MDS population through an extensive, multidimensional analysis of social media posts. By combining disease-specific search logic, advanced NLP, and psychographic profiling, we provide actionable insights that can inform physician education, patient communication strategies, and supportive care programs tailored to the HR-MDS community.

### Limitations

This study used data collected from various electronic sources available publicly on the internet. Although the analysis of this data can lead to powerful and actionable insights, this study also acknowledges the limitations arising from such data usage.

#### Lack of Representativeness

The population of patients with MDS and their caregivers using the internet for research is limited to those who choose to engage in social media. Therefore, this study implicitly excluded any patients with MDS or caregivers who do not have access to the internet or decided not to engage with the social media platforms from which we collected data.

#### Geographical Bias

The objective of this study was to capture the experience of patients with MDS and their caregivers in the English language. This very choice limits the scope of this study to the regions of the world that use the English language.

#### Active Engagement Bias

An unknown proportion of the users of web-based resources are likely to be passive consumers. The experiences of this segment of the population of patients with MDS and caregivers may not necessarily be reflected in the data used for this study.

#### Lack of Heterogeneity

Health care systems vary widely across even English-speaking regions, as do the experiences of patients with MDS and their caregivers. This study does not claim to capture this heterogeneity across various geopolitical entities accurately.

#### Missing Data

It is anticipated that not all patients or caregivers posted all variables listed in the variable table.

#### Variable Longitudinality

There were substantial variations in the frequency of posts. In those cases, themes and sentiment could only be determined at snapshots along the patient’s journey.

### Comparison With Prior Work

Existing literature on MDS has largely concentrated on clinical and pathological dimensions, such as risk stratification, treatment regimens, and survival outcomes. Although there has been increasing recognition of the psychosocial burden faced by patients, most prior studies have addressed this in a fragmented or secondary manner, without systematically examining the emotional and experiential aspects across the patient journey. Notably, the specific needs and perspectives of patients with HR-MDS remain underexplored in current patient-reported outcomes research. In contrast, this study provides a structured analysis of patient and caregiver sentiment, identifying consistent emotional themes and their contextual drivers. By integrating these insights into a broader framework of the MDS experience, our work contributes a novel, patient-centered perspective that complements the clinical discourse and highlights actionable opportunities for improving quality of care and patient engagement.

### Conclusions

Patients with MDS and caregivers predominantly experience concern, frustration, anxiety, confusion, and fear. Concern stems from apprehensions about blood counts, disease burden, and quality of life. Frustration arises due to perceived inadequacies and complexities in treatment and management, while anxiety is linked to health deterioration and treatment outcomes. Confusion is attributed to the difficulty in comprehending the nature of MDS and its management. Uncovering and mapping underlying themes with sentiments along the journey of patients with MDS can inform areas of need for patient-centered care and the development of patient-focused solutions with the potential to improve the patient experience. Ongoing exploratory research includes focusing on patients with HR-MDS and specific related issues. Findings from this study can inform areas of need for patient-centered care for HR-MDS and the development of patient-focused solutions with the potential to improve the patient experience.

## Abbreviations

AML: acute myeloid leukemia
HR-MDS: high-risk myelodysplastic syndrome
MDS: myelodysplastic syndrome
ML: machine learning
NLP: natural language processing
